# The KT Jeang Retrovirology prize 2015: Paul Bieniasz

**DOI:** 10.1186/s12977-015-0208-y

**Published:** 2015-10-05

**Authors:** Retrovirology Editorial

**Affiliations:** BioMed Central, 236 Gray′s Inn Road, London, WC1X 8HB UK


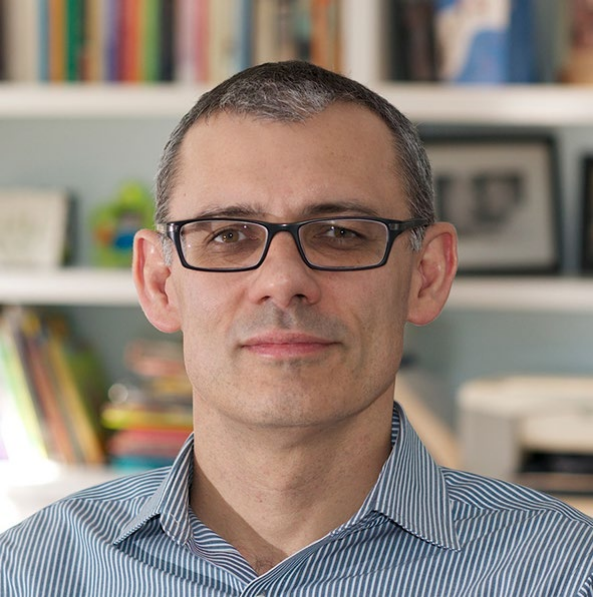
Paul Bieniasz graduated from the University of Bath, UK in 1990, and began his career in retrovirology with Jonathan Weber and Myra McClure at St. Mary’s Hospital Medical School in London. Initially, he worked HIV-1 entry, and the early development of PCR-based assays to quantify HIV-1 burden in patients [1]. For his Doctoral degree, Bieniasz shifted his focus and worked with McClure on the foamy viruses. In particular, he characterized novel foamy virus isolates from apes, in so doing demonstrating a close relationship between ‘human’ and chimpanzee viruses [2]. He also developed some of the first foamy virus-based gene transfer vectors [3] and showed that foamy virus infection was dependent on cell division [4].

After graduating, Bieniasz joined Bryan Cullen at Duke University as a postdoctoral fellow, from 1996 to 1999, and returned to HIV-1 research. For his first set of experiments, he exploited sequence differences between the newly identified human and mouse CCR5 proteins to determine sites that were key for strain-dependent interactions with the HIV-1 envelope [5]. In a second set of studies he again used functional differences between human and proteins to show that a single amino-acid difference in the cyclin T1 underlay the differential ability of Tat:P-TEFb complexes to bind TAR and, thus, the species-dependent activity of HIV-1 Tat [6]. Bieniasz and Cullen also made a key finding that artificial P-TEFb recruitment to a promoter proximal RNA element was sufficient to stimulate that transcriptional elongation activity in the absence of Tat [7].

Bieniasz started his own lab at the Aaron Diamond AIDS Research Center and Rockefeller University in late 1999. Since then he has worked on many and varied aspects of retrovirus replication. Initially, building on work that he began with Cullen, Bieniasz found that rodent cells engineered to express human CD4, CCR5 and cyclinT1, could support early but not late steps in HIV-I replication [8]. His characterization of these novel host range restrictions sparked a long-standing interest in HIV-1 assembly and budding. Indeed, among the Bieniasz lab’s early findings was the demonstration that the matrix domain of Gag exerted an auto-inhibitory effect on Gag-membrane interactions that contributed to the apparent block to HIV-I assembly in rodent cells [9, 10].

A key set of studies from the Bieniasz lab helped to elucidate the mechanisms by which so-called ‘late-budding’ domains enable enveloped virus particle release. Specifically, Bieniasz and colleagues contributed to the discoveries of key roles for Tsg101, ALIX and HECT-ubiquitin ligases and ubiquitin in the budding of HIV-1, Ebola and other viruses [11–14]. These proteins interacted with numerous components of the then newly discovered ESCRT pathway which the Bieniasz lab showed were important for retrovirus particle release [12, 15, 16].

For a time, the subcellular location at which HIV-1 particle assembly occurs was controversial. The Bieniasz lab resolved this question, demonstrating clearly that it occurs at the plasma membrane [17]. Building on that work, Bieniasz collaborated with Sanford Simon to develop imaging techniques that, for the first time, allowed the genesis of individual virus particles to be visualized in living cells [18]. This advance enabled unprecedented studies of the dynamics of the assembly and budding of individual HIV-1 virions. In particular, elaborations of this technique allowed Bieniasz and Simon to visualize and quantify the dynamics of viral genomic RNA movement and encapsidation [19], as well as the recruitment of ESCRT proteins to sites of virion release [20].

More recently, the Bieniasz lab developed new biochemical and crosslinking-nextgen sequencing approaches to reveal, in unprecedented detail, how viral proteins and RNA interact during particle assembly [21, 22]. This new work has redefined the rules that govern how HIV-1 packages its genome, and suggests that the unusual A-rich nucleotide composition of the HIV-1 genome helps to drive viral RNA interaction with Gag molecules as they assemble into virions [22]. These new approaches have also uncovered a striking and specific interaction between the HIV-1 matrix domain and tRNA, specifically in the infected cell cytoplasm, that may contribute to the ability of HIV-I matrix to auto inhibit, and thereby delay, HIV-1 virion assembly [22, 23].

A second major area of interest for Bieniasz has been the discovery and characterization of intrinsic and innate cellular antiviral defenses. A significant part of this work has been done with his wife and colleague, Theodora Hatziioannou. The Bieniasz lab’s first work in this area revealed that primate cells possessed an antiviral activity that could block HIV-I infection at a post entry step, targeting the incoming viral capsid [24]. Notably, the specificity of this novel antiviral activity varied dramatically in a species-dependent manner and could inhibit very diverse retroviruses [25, 26]. The protein responsible for this activity was later identified (by the Sodroski lab) as TRIM5, and the Bieniasz lab performed key studies of its activity against diverse retroviruses [27], mapped determinants of specificity in the viral capsid and in TRIM5 [28, 29], and provided insights into TRIM5’s mechanism of action [30, 31].

Bieniasz’s interests in HIV-1 assembly and in antiviral proteins have sometimes overlapped. For example, his group showed that RNA recruited APOBEC3 into virons through apparently sequence-nonspecific interactions [32]. A seminal contribution by the Bieniasz lab was a collection of studies on the HIV-I Vpu protein. Initially, they showed that Vpu antagonized the action of an unknown interferon-induced protein that could apparently tether divergent enveloped viruses at the surface of infected cells [33, 34]. These studies led directly to the discovery of Tetherin [35], and a series of papers on Tetherin function. For instance, The Bieniasz lab showed that Tetherin could inhibit the release of remarkably diverse viruses [36] and generated a Tetherin knockout mouse to demonstrate the antiviral action of Tetherin in vivo [37]. Bieniasz’s group also delineated the molecular mechanism by which Tetherin inhibits particle release, demonstrating that Tetherin inserted itself into the lipid envelope of virions to cause their entrapment, and that its overall protein structure and not primary sequence are required for activity [38, 39]. In other studies with Hatziioannou, Bieniasz showed that SIVs lacking a Vpu protein often employ another viral accessory protein, Nef, as a Tetherin antagonist [40, 41]. Bieniasz and colleagues found that both of these viral antagonists work in a host species restricted manner [40, 42], as governed by Tetherin sequence variation They also revealed key aspects of the molecular mechanisms by which these viral antagonists function [43, 44].

The finding that an interferon-induced protein could directly inhibit HIV-1 replication inspired Bieniasz to search, in collaboration with his colleague Charles Rice, for additional interferon induced genes (ISGs) that might contribute to the antiretroviral activity of interferons [45, 46]. One result of this search was the co-discovery that Mx2 exhibits anti-HIV-1 activity during the post entry/preintegration steps of HIV-1 replication [47]. The Bieniasz group also showed that Mx2 apparently blocks capsid-dependent entry of HIV-1 preintegration complexes into the nucleus, and exhibits signatures of diversifying selection in it N-terminal domain that governs nuclear pore localization and antiviral activity and specificity [47, 48].

By exploiting knowledge of specific host-range restrictions imposed by antiviral proteins, Bieniasz, Hatziioannou and their collaborators, Jeff Lifson and Vineet KewalRamani have engineered HIV-1 to overcome barriers to HIV-1 replication in monkeys [49], allowing the generation of new animal models of HIV-I infection [50]. In particular, Bieniasz and Hatziioannou identified a second example of a TRIM5-CypA fusion protein in pig-tailed macaques that, remarkably, could not inhibit HIV-1 infection [51]. This discovery enabled the use of viral engineering and adaptation to develop an HIV-1 strain that, for the first time, can cause AIDS in a non-hominid species [52]. This team has also devised a procedure for generating pathogenic SHIVs that promises to expand the range of challenge viruses available for HIV-I vaccine studies [53].

In addition to these core interests, Bieniasz has a broad interest in the function and evolution of a range of viral and host proteins that are involved in retrovirus replication [54–58]. The Bieniasz group has also pioneered the field of paleovirology. His group showed that an extinct retrovirus (HERV-K) could be resurrected in functional form from molecular fossils that are present in modern genomes [59] and uncovered evidence of ancient interactions between APOBEC3 proteins and retroviruses in the form of hypermutated endogenous proviruses in humans and chimpanzees [60, 61]. They also completed the first identification of an entry receptor for a presumptively extinct virus (CERV-2) using a reconstituted ancestral envelope protein [62].

Bieniasz has served on several review and advisory board including the NIH AIDS Molecular and Cellular Biology study section (2004–2009) including as Chair (2007–2009) and on the NCI Board of Scientific Counselors (2010–2014). He has been an investigator of the Howard Hughes Medical Institute since 2008. Bieniasz was a 2003 recipient of the Elizabeth Glaser Scientist Award from the Elizabeth Glaser Pediatric AIDS Foundation and the 2010 recipient of the Eli Lilly and Company Research Award. He was elected to the American Academy of Microbiology and received an NIH MERIT award in 2011, and was awarded the Ohio State University Center for Retrovirus Research Distinguished Career award in 2015.
